# Genetic Study of Early Onset Parkinson’s Disease in Cyprus

**DOI:** 10.3390/ijms232315369

**Published:** 2022-12-06

**Authors:** Rana Abu Manneh, Paraskevi P. Chairta, Ellie Mitsi, Maria A. Loizidou, Andrea N. Georgiou, Yiolanda P. Christou, Marios Pantzaris, Eleni Zamba-Papanicolaou, Andreas Hadjisavvas

**Affiliations:** 1 Neuroepidemiology Department, The Cyprus Institute of Neurology and Genetics, 2371 Nicosia, Cyprus; 2 Cancer Genetics, Therapeutics & Ultrastructural Pathology Department, The Cyprus Institute of Neurology and Genetics, 2371 Nicosia, Cyprus; 3 Department of Hygiene and Epidemiology, Medical School, University of Ioannina, 45110 Ioannina, Greece; 4 Acropoleos Medical Center, 2013 Nicosia, Cyprus; 5 Neuroimmunology Department, The Cyprus Institute of Neurology and Genetics, 2371 Nicosia, Cyprus

**Keywords:** early onset Parkinson’s disease (EOPD), genetic investigation, Greek-Cypriot Parkinson’s patients

## Abstract

Parkinson’s Disease (PD) is a multifactorial neurodegenerative disease characterized by motor and non-motor symptoms. The etiology of PD remains unclear. However, several studies have demonstrated the interplay of genetic, epigenetic, and environmental factors in PD. Early-onset PD (EOPD) is a subgroup of PD diagnosed between the ages of 21 and 50. Population genetic studies have demonstrated great genetic variability amongst EOPD patients. Hence, this study aimed to obtain a genetic landscape of EOPD in the Cypriot population. Greek-Cypriot EOPD patients (*n* = 48) were screened for variants in the six most common EOPD-associated genes (*PINK1*, *PRKN*, *FBXO7*, *SNCA*, *PLA2G6*, and *DJ-1*). This included DNA sequencing and Multiplex ligation-dependent probe amplification (MLPA). One previously described frameshift variant in *PINK1* (NM_032409.3:c.889del) was detected in five patients (10.4%)—the largest number to be detected to date. Copy number variations in the *PRKN* gene were identified in one homozygous and 3 compound heterozygous patients (8.3%). To date, the pathogenic variants identified in this study have explained the PD phenotype for 18.8% of the EOPD cases. The results of this study may contribute to the genetic screening of EOPD in Cyprus.

## 1. Introduction

Parkinson’s Disease (PD) is the second most common neurodegenerative disease worldwide [1]. By 2016, the global population of PD had reached 6 million [2], and by 2020, it has been estimated to have increased to 9.4 million (95% CI) [3]. In a study investigating the global burden of PD, gender stratification revealed that the prevalence is higher in males than females with a ratio of 1 to 1.36–1.43 [2]. PD can be stratified into three subgroups based on the age of disease onset; Juvenile-onset, Early-onset (EOPD), and Late onset (LOPD). EOPD is diagnosed between the ages of 21 and 50 [4], and accounts for 10–15% of PD cases [5]. The majority of PD cases are sporadic, and report no previous family history of the disease [6]. Whereas the familial PD cases include inherited or monogenic forms of the disease [6]. Interestingly, Ferguson et al., as well as other groups have demonstrated that there is no significant difference between the percentage of familial cases in EOPD and LOPD [7,8].

Pathologically, PD is characterized by two major processes; premature loss of dopamine-producing neurons in the substantia nigra pars compacta (SNpc), and abnormal deposition of Alpha-Synuclein protein in the brain and spinal cord in the form of Lewy bodies (LBs) and Lewy neurites (LNs) [9,10]. The resultant dopamine deficiency in the brain leads to a broad range of motor and non-motor manifestations. However, this occurs only after 70–80% of dopaminergic neurons are lost [11]. Thus, by the time PD is diagnosed, the disease has already manifested and irreversible deterioration of the nervous system has taken place [12].

Thirty years ago, the development of PD was supposed to be a result of environmental exposures, such as contact with pesticides, prior head trauma, and well-water drinking [13]. Nevertheless, more recent studies indicate that a complex interplay between environmental and genetic factors contributes to the triggering of the disease [14]. By 2018, 15 genes with disease-causing mutations have been identified to cause 30% of the familial cases, and 3–5% of the sporadic PD cases [15,16]. As described in a review by Blauwendraat et al., rare variants in more than 20 genes have been reported to cause PD, with varying degrees of penetrance [17]. The genes most commonly associated with EOPD are Alpha-Synuclein (*SNCA*), Parkin (*PRKN*), Phosphatase and Tensin Homolog-induced Putative Kinase 1 (*PINK1*), Protein Deglycase DJ-1 (*DJ-1*), Phospholipase A2 (*PLA2G6*), and F-Box Only Protein 7 (*FBXO7*) [17]. It is important to note that there is an overlap between certain genes that contain disease-causing mutations and those that harbor risk-variants associated with PD [18,19]. By definition, autosomal recessive (AR) inheritance suggests that the two gene alleles must be mutant for the disease to be clinically apparent, yet this is not always the case in PD [19]. Heterozygous variants in EOPD-associated genes with AR inheritance, such as *PRKN* and *PINK1* have been reported to increase PD risk, possibly due to compound heterozygosity or partial loss-of-function [19]. The function of the EOPD-associated genes has been linked to protection against mitochondrial dysfunction, as well as the mediation of mitophagy (programmed autophagy of damaged mitochondria) [20,21,22]. Other functions include roles in synaptic transmission [23,24] and phospholipid remodeling [25,26,27].

Single nucleotide variants (SNVs), insertion or deletion (Indels) and copy number variations (CNVs) in PD-associated genes have been detected across many populations and ethnic groups [5,27,28,29,30,31,32,33,34,35,36,37,38,39,40,41,42,43,44,45,46,47,48,49]. CNVs in *PRKN* have been reported in several population studies and account for up to 12% of PD cases [31,33,34,39,42,43,45,46,47,48,49], with unique exceptions observed in certain ethnic groups [39]. CNVs have also been reported less frequently in *SNCA*, *PINK1* and *DJ-1* [31,34,36,38,50,51,52]. Previous population studies have shown great genetic variability in the detected PD-associated variants, suggesting that geographic location and ethnic origin influence the detection outcome [30]. Inclusivity is very important in PD research and hence filling the genetic gap in underrepresented populations will be very useful for better disease understanding [53].

Therefore, the aim of this study was to analyze the genetic landscape of EOPD in the Cypriot population for the first time to date. Moreover, we wanted to investigate whether there were any novel or recurring variant(s) in our population. For that purpose, DNA sequencing and copy number analysis of the most common EOPD-associated genes was performed.

## 2. Results

### 2.1. Cohort Description

This study included 48 EOPD patients with a median age of onset (AOO) of 46 years (range 23–50 years). Males had a higher frequency of EOPD compared to females (M:F ratio, 1.67:1) in this cohort. Family history was recorded for 18 (37.5%) patients in total; first- and second-degree relatives with PD were reported for 13 (27.1%) and 5 (10.4%) patients, respectively. Among the familial cases were 2 sets of siblings; dizygotic female twins and one set of male and female siblings.

### 2.2. Pathogenic Variants

Pathogenic variants were identified in 9 out of the 48 patients tested (18.8%) (Table 1). Of these, 5 (10.4%) patients carried the same frameshift mutation in the *PINK1* gene, and 4 (8.3%) had CNVs in the *PRKN* gene. Among the carriers of the p.(Asp297Metfs*22) frameshift mutation in *PINK1*, 3 were homozygous and 2 were heterozygous. Out of the patients with CNVs in *PRKN*, one was a homozygous carrier of the deletion in exons 3 and 4, whereas 3 patients were compound heterozygous carriers. Overall, 6 out of the 18 patients with a family history (33.3%), and 3 out of the 30 patients without a family history of PD (10%) were carriers of a pathogenic mutation. The clinical characteristics of those patients are demonstrated in Table 2.

### 2.3. Variants of Uncertain Significance (VUS)

In this study, 2 variants with conflicting interpretations of pathogenicity in ClinVar [54] were identified; c.1372A>C (p.Met458Leu) in *PRKN*, and c.416G>A (p.Arg139His) in *PLA2G6*. The aforementioned variants were detected in two male patients, with AOO = 49 and AOO = 50, respectively. Both patients had no family history of PD, and had no other pathogenic variants in the genes under investigation in this study. The former variant, rs182893847 [55], has been reported in HGMD [56] as a likely disease-causing mutation but with questionable pathogenicity (CM096654, DM?), and was described to have a rare global frequency in the 1000 Genome Project (0.0014) [57]. The latter variant, rs141825182 [55], has not been reported by neither HGMD nor 1000 Genome Project, but was described by ALFA [58] to be very rare worldwide (0.0017). According to the ACMG guidelines [59], both variants are classified as ‘variants of uncertain significance (VUS)’. However, according to the Invitae Sherloc variant classification [60], a refinement of the ACMG guidelines, both variants are classified as ‘likely benign’.

## 3. Discussion

To the best of our knowledge, this is the first genetic study conducted on Greek-Cypriot EOPD patients targeting EOPD-associated genes. This study is representative of the Greek-Cypriot population as patient recruitment occurred at a tertiary center where the majority of EOPD cases in Cyprus are being referred to. Given this information, the capture rate is expected to be >90%. The female-to-male ratio of EOPD patients in the present study was slightly higher than in previous reports [2].

We report 9 patients (18.8%) in our cohort that were positive for a pathogenic variant (Table 1). Of those, 56% harbored the same single nucleotide deletion in *PINK1*, and 44% were carriers of CNVs in *PRKN.* Variants in *PRKN* are more common than *PINK1* amongst EOPD patients from European and Asian populations [5,30,33]. For example, in a Dutch cohort, the frequency of *PRKN* variants (6%) was greater than *PINK1* (1%) [34]. Similarly, in an Irish cohort, 6.9% of EOPD cases were carriers of a *PRKN* pathogenic variant, while no variants were detected in *PINK1* [37]. In a study on EOPD in central European populations (Czech, German, Polish, and Ukrainian), *PINK1* pathogenic variants were only reported in 0.6% of the Polish cohort, whereas *PRKN* variants were detected in all 4 cohorts (2.6–9.1%) [5]. Furthermore, in a large multicentre study including Caucasian (French and Turkish), Arab-Berber and other ethnicities, *PRKN* mutations (12.5%) were more frequent than *PINK1* (1.9%) [33]. In contrast to the above population studies, we had a 10.4% frequency of *PINK1* mutation and 8.3% of *PRKN*. Interestingly, *PINK1* and *PRKN* mutation frequencies in our Greek-Cypriot cohort are more similar to Arab-Berbers (*PINK1* = 6.4%, *PRKN* = 7.5%) than Caucasians (*PINK1* = 0.9%, *PRKN* = 13.5%) [33].

The frequency of pathogenic variants amongst cases with a known family history of PD in this study (33%) compared to those without (10%), is similar to previous reports [16,33]. It is important to note that there might be a recall bias amongst the sporadic cases regarding family history of PD.

No pathogenic variants were detected in the other 4 genes (*DJ-1*, *SNCA*, *PLA2G6* and *FBXO7*) under investigation in this study. The majority of EOPD cohorts either report a very small frequency of pathogenic variants in *DJ-1* (<2%) [33,34,36,50], or similar to our study- not at all [5,37]. Unlike other groups [31,34,47], we did not detect any missense variants or CNVs in the *SNCA* gene. Pathogenic variants in *PLA2G6* and *FBXO7* are very rare [30], and have not been detected in our cohort.

*PINK1* loss-of-function variants cause AR-EOPD, and the presence of biallelic variants may result in a dysfunctional kinase domain and/or mitochondrial motif encoded by the gene. We identified the largest number of patients (*n* = 5) carrying the p.(Asp297Metfs*22) pathogenic frameshift variant in exon 4 of *PINK1*, as compared with 3 carriers reported worldwide [61,62]. Savettieri et al., first described the c.889del single nucleotide deletion in a consanguineous Sicilian family; they reported 2 EOPD siblings (male AOO = 28, male AOO = 29) homozygous for the frameshift variant [61]. Kumazawa et al., reported the same deletion in a Greek female with juvenile-onset (AOO = 10), an offspring of a consanguineous marriage with a first-degree family history of PD (affected sister) [62]. The single nucleotide deletion leads to the premature termination of the polypeptide kinase domain and by that the loss of the PINK1 protein function.

Out of the 5 *PINK1* variant carriers in this study, 2 were dizygotic twin sisters, both homozygous carriers (Table 1, case no. 1–2). However, they had a difference of 23 years in their AOO, as well as a differential clinical presentation; one (AOO = 23) was wheelchair-bound by the age of 65, had depression and cognitive decline. Whereas the other sister (AOO = 46) presented with mild motor symptoms, and 20 years after her onset is still at stage 1 of the Hoehn & Yahr scale (Table 2). It is important to note that the mildly affected sister might have had an earlier disease onset than 46. The aforementioned patient was only seen in the clinic after the kinship with her twin sister came to the attention of the neurologist; therefore, early diagnosis might have been lost due to this mild form of PD. The third homozygous carrier of the frameshift variant (Table 1, case no. 3) developed PD symptoms at the age of 41 and reported no family history, had hypertension and depression. The differential AOO, disease progression, and clinical presentation amongst the patients with same homozygous pathogenic variant may be credited to highly penetrant rare variants being affected by other genetic and environmental factors [17]. These cases highlights the complex and multifactorial nature of PD.

The frameshift variant could explain the EOPD phenotype in the homozygous patients (*n* = 3). However, since *PINK1* is a recessive gene, the heterozygous carriers (*n* = 2) might be harboring other risk variants not yet identified. Nevertheless, heterozygous variants in the AR PD-associated genes, such as *PRKN* and *PINK1*, have been previously reported to possibly increasing PD susceptibility [19,63]. However, the mechanism by which these variants affect disease development is still unclear. Epigenetic inactivation of the wild type allele, creating a pseudo-dominant effect of the heterozygous variant, could be a possible explanation [64]. Alternatively, an EOPD phenotype in heterozygous carriers can be explained by the co-occurrence of heterozygous variants in more than one AR EOPD-associated gene [64,65]. Digenic inheritance in EOPD has been previously reported in cases with heterozygous variants in *PINK1* and *DJ-1* [64], *PINK1* and *PRKN* [65], and *PRKN* and *SNCA* [66]. Similarly, the identified heterozygous carriers in this cohort could be harboring other heterozygous variants in genes not investigated in this study.

*PRKN* variants are another cause of AR-EOPD due to loss-of-function of the protein product. The MLPA analysis revealed 4 patients (8.3%) in the Cypriot EOPD cohort with CNVs in the *PRKN* gene. These findings are aligned with previous population studies that reported similar percentages (<12%) [31,34,35,38,40,41,42,45,47,48]. One patient was homozygous and three were compound heterozygous for CNVs in *PRKN* (Table 1, case no. 6–9). For the homozygous carrier, we were unable to amplify exons 3 and 4 with Sanger sequencing which further verifies the biallelic deletion. Regarding the compound heterozygous patients, two report non-adjacent exon deletions. Compound heterozygous carriers of CNVs in the *PRKN* gene have been reported in the past; heterozygous deletions of non-adjacent exons [48], as well as heterozygous deletion of one exon and duplication of another [31]. In addition, we were able to confirm that 2 out of the 4 *PRKN* variant carriers (Table 1, case no. 6–7) developed cancer several years (25 and 6 years, respectively) after their PD symptom onset. Specifically, the patients that had at least one biallelic exon deletion, developed cancer. In addition, the father of one of the patients that did not develop cancer to date (Table 1, case no. 8), died of Leukemia at the age of 58. However, since paternal samples were not collected in this study, we could not confirm the pattern of inheritance. The role of *PRKN* in cancer, as a tumor suppressor gene, has been previously discussed [67,68,69].

Even though *SNCA* variants account only for a minority of PD cases globally, the disease pathology involving the *SNCA* protein product seems to be shared amongst the majority of PD patients [70]. One of the hallmarks of PD is the abnormal deposition of α-synuclein in the central nervous system in the form of LBs and LNs [70]. Besides missense variants and CNVs in *SNCA*, α-synuclein aggregation could be a result of abnormal protein clearance, impaired mitochondrial function, and increased oxidative stress that may induce proteins to alter their structure [71]. Consequently, LBs may contribute to PD by disrupting dopamine regulation in DA neurons leading to its toxic concentration within the cell [72]. Alternatively, LBs can disrupt the ubiquitin-proteasome (UPS) clearance system and by that alter cellular homeostasis [72]. α-synuclein aggregates bind with high affinity to mitochondria, inhibiting mitochondrial protein uptake and promoting reactive oxygen species (ROS) formation [73]. ROS may damage the mitochondrial DNA and have a positive feedback-loop effect on α-synuclein aggregation [73]. Furthermore, Shimura et al., hypothesized that a functional interaction might exist between the *SNCA* and *PRKN* proteins; Loss-of-function variants in *PRKN* might result in Parkin not being able to effectively ubiquitinate the α-synuclein protein, hence its intracellular accumulation [74].

Additionally, DJ-1, a multifunctional protein that is involved in oxidative stress sensing and chaperonal activities, has been reported to directly interact with α-synuclein monomers and oligomers [75]. DJ-1 overexpression was shown to decrease the dimerization of α-synuclein, while mutant forms of the protein impaired this observation [75]. PINK1, Parkin and DJ-1 polypeptides have been identified to form a ligase complex that aims to ubiquitinate and degrade non-folded proteins [76]. Parkin, encoded by *PRKN*, has been reported to play a role downstream of PINK1 in the mediation of mitophagy. The PINK1 kinase has been directly seen to phosphorylate Parkin and cause its localization to mitochondrial outer membranes [22]. Once activated, Parkin-a ubiquitin-E3 ligase, is able to ligate ubiquitin molecules onto its mitochondrial substrates [77], and by that promote the removal of dysfunctional mitochondrial proteins and the autophagy of damaged mitochondria [78]. Furthermore, FBXO7 has been reported to directly interact with PINK1 and Parkin and play a role in the Parkin-mediated mitophagy process [79]. The EOPD-associated genes that were under investigation in our study seem to interact with each other on a structural and functional level. Hence, variants in either one of the aforementioned genes have the potential to alter cellular processes and potentially increase PD susceptibility.

Currently, the diagnosis of PD is clinical and based on the presence of motor features. Early onset patients have a challenging journey towards a PD diagnosis as their initial symptoms may vary and their young age of onset may lead to differential diagnoses. To date, 18.8% of the 48 tested Cypriot patients had a clear monogenic cause for their PD phenotype. Almost 1 in every 5 patients in our cohort has been identified as a carrier of either a *PINK1* or *PRKN* variant. Hence, the results of this study may contribute to the genetic screening of EOPD in Cyprus.

Some limitations of this study were that we did not include all PD-associated genes in Sanger sequencing. However, the MLPA analysis included probes for several additional genes associated with PD, including ATPase 13A2 (*ATP13A2*), Leucine-rich repeat kinase 2 (*LRRK2*), GTP Cyclohydrolase 1 (*GCH1*) and Ubiquitin C-terminal hydrolase L1 (*UCHL1*). Another limitation of this study was that we used a candidate gene approach while other population studies used genome wide association studies that gave a broader range of results. Even though we had quite a high detection of monogenic forms of EOPD, the idiopathic cases still account for >80%. Overcoming the limitations of this study could potentially decrease this percentage. Future work ought to include whole exome sequencing for the idiopathic EOPD cases. Genetic analysis and determining familial aggregation of a LOPD Cypriot cohort would be helpful for future comparison with this study’s EOPD cohort. This will allow for a more inclusive genetic view of PD in Cyprus.

Even though Cyprus is officially part of the European continent, its geographic location and historical events reflect how it might be integrated in the Middle East, giving rise to its unique genetic profile. Thus, the importance of this study is the addition of another piece to the puzzle of PD genetics and the reduction of the research gap in underrepresented populations [53,80].

## 4. Materials and Methods

### 4.1. Participants

A cohort of 48 EOPD patients of Greek-Cypriot origin was recruited from the neurology clinics of the Cyprus Institute of Neurology and Genetics (CING) in Nicosia, Cyprus. Since it is a tertiary referral center for genetic and neurological disorders in Cyprus, most EOPD patients in Cyprus are referred to CING by their physicians, and the majority are being followed up there. All patients were evaluated by CING neurologists and fulfilled the inclusion criteria; (i) clinical diagnosis of PD, and (ii) age of onset between 21–50 years. Patients with additional neurological symptoms were excluded from the study. EOPD patients were recruited from 2014 until 2021. The study was approved by the Cyprus National Bioethics Committee and conducted in accordance with the 1964 Declaration of Helsinki. Informed consent was obtained from all patients.

### 4.2. Genetic Analysis

#### 4.2.1. DNA Sequencing

Genomic DNA was extracted from peripheral blood lymphocytes using standard methods. Primers were designed for the 6 most common EOPD-associated genes; *SNCA* (NM_000345.4), *PRKN* (NM_004562.3), *PINK1* (NM_032409.3), *DJ-1* (NM_007262.5), *PLA2G6* (NM_003560.4) and *FBXO7* (NM_012179.4). Primer sequences and PCR conditions can be provided upon request. The Sanger sequencing method was used for the amplification and sequencing of the coding exons and flanking intronic regions of the genes. The samples were run in an ABI 3130xl genetic analyzer (Applied Biosystems, Waltham, MA, USA) using a 36 cm capillary (Applied Biosystems, Waltham, MA, USA), and POP-7 Performance Optimized Polymer (Applied Biosystems, Waltham, MA, USA). Data analysis was carried out using the Sequencing Analysis software (Applied Biosystems, Waltham, MA, USA). The reference genomic, transcript, and protein sequences used to describe the sequence variants were obtained from RefSeq: NCBI Reference Sequence Database https://www.ncbi.nlm.nih.gov/refseq/ (accessed on 18 June 2020). The accession numbers and classifications of previously known variants were retrieved from ClinVar [54], Human Gene Mutation Database (HGMD^®^) [56], dbSNP [55], and the literature. Alternative allele frequencies (AAF) were retrieved from the Allele Frequency Aggregator (ALFA, Release Version: 20200227123210) [58], and the 1000 Genome Project [57].

#### 4.2.2. Detection of Copy Number Variation (CNV)

Patients were screened for CNVs using the Multiplex Ligation-Dependent Probe Amplification (MLPA) method. The SALSA^®^ MLPA^®^ Probemix P051-D2 and P052-D2 Parkinson kits (MRC-Holland, Amsterdam, The Netherlands) were applied according to the manufacturer’s instructions. The Coffalyser.Net software version 220513.1739 (MRC-Holland, Amsterdam, The Netherlands) was used for data analysis.

### 4.3. In Silico Predictions

In silico tools were applied to predict the downstream effect of the variants detected in our study that have not been previously reported in ClinVar or have conflicting interpretations of pathogenicity, as recommended by the American College of Medical Genetics and Genomics (ACMG) standards and guidelines [59]. Missense prediction tools were utilized to determine the effect of the coding variants on the amino acid sequence, and hence the protein structure and function. While splice site prediction tools were implemented to predict if the variants had any effect on splicing.

## 5. Conclusions

In conclusion, 18.8% of the Greek-Cypriot EOPD population had a clear monogenic cause for their PD phenotype. One previously described frameshift variant in *PINK1* (NM_032409.3:c.889del) was detected in five patients (10.4%), and CNVs in the *PRKN* gene were identified in four patients (8.3%). Almost 1 in every 5 patients has been identified as a carrier of either a *PINK1* or *PRKN* pathogenic variant. Hence, the results of this study may contribute to the genetic screening of EOPD in Cyprus.

## Figures and Tables

**Table 1:** Pathogenic variants identified in this study.

Case No.	RefSeq Gene	Sequence Variant	Copy Number Variant	AOO	Gender	Known Family History of Disease	Affected Family Member, AOO
**1**	*PINK1* ^1^	c.889del	*_*	46	F	Yes	dizygotic twin sister, 23
		p.(Asp297Metfs*22) (hom)					
**2**	*PINK1* ^1^	c.889del	*_*	23	F	Yes	dizygotic twin sister, 46
		p.(Asp297Metfs*22) (hom)					
**3**	*PINK1* ^1^	c.889del	*_*	41	F	No	*_*
		p.(Asp297Metfs*22) (hom)					
**4**	*PINK1* ^1^	c.889del	*_*	50	F	Yes	father, unk
		p.(Asp297Metfs*22) (het)					
**5**	*PINK1* ^1^	c.889del	*_*	43	M	No	*_*
		p.(Asp297Metfs*22) (het)					
**6**	*PRKN* ^2^	*_*	Ex3–4del (hom)	45	M	Yes	grandmother, unk
**7**	*PRKN* ^2^	*_*	Ex2del (het); Ex2–4del (het)	50	F	Yes	sister, 56
**8**	*PRKN* ^2^	*_*	Ex2del (het); Ex5del (het)	35	M	No	*_*
**9**	*PRKN* ^2^	*_*	Ex3–4del (het); Ex6–7del (het)	50	F	Yes	father, unk

RefSeq, Reference Sequence database built by the National Center for Biotechnology Information; AOO, age of onset; hom, homozygous; het, heterozygous; Ex, exon; del, deletion; unk, unknown; ^1^ NM_032409.3, ^2^ NM_004562.3.

**Table 2:** Clinical characteristics of patients carrying a pathogenic variant.

Case No.	RefSeq Gene	Clinical Symptoms at Onset	MDS-UPDRS Score Total (Parts I, II, III, IV)	H & Y	Years After Onset ^3^	Dyskinesia	Surgery	Other Diseases, AOO
**1**	*PINK1* ^1^	R, B	16	1	20	No	No	hypothyroidism, 59;
			(1, 4, 11, 0)					hypertension, unk;
**2**	*PINK1* ^1^	T, PI	189	5	44	Yes	No	dysarthria, 54;
								cognitive decline, 54;
			(34, 45, 99, 11)					depression, unk
**3**	*PINK1* ^1^	T, R, PI	111	5	35	Yes	STN DBS	dementia, 80;
								depression, 41;
			(23, 28, 60, 0)					arrhythmia, unk;
								hypertension, unk
**4**	*PINK1* ^1^	R, B	36	1	12	No	No	*_*
			(14, 10, 11, 1)					
**5**	*PINK1* ^1^	R, B	94	3	23	Yes	STN DBS	benign prostatic hyperplasia, unk
			(13, 31, 38, 12)					
**6**	*PRKN* ^2^	T, R, B	76	3	24	Yes	No	colon cancer, 75;
								skin melanoma with lung/liver metastasis, 70;
								depression, 55;
			(18, 14, 36, 8)					memory disturbances, 55;
								CVD, unk;
								hypertension, unk
**7**	*PRKN* ^2^	T	43	2	11	Yes	No	sleep disturbances, 68;
								breast cancer, 56;
								depression, 50;
			(19, 11, 13, 0)					hypertension, 41;
								diabetes, unk
**8**	*PRKN* ^2^	R, B	22	1	3	Yes	No	bipolar disorder, 41;
			(8, 3, 11, 0)					depression, 33
**9**	*PRKN* ^2^	R	43	1	13	No	No	hypercholesterolemia, unk
			(8, 7, 28, 0)					hypertension, unk

RefSeq, Reference Sequence database built by the National Center for Biotechnology Information; MDS-UPDRS, Movement Disorder Society-Sponsored Revision of the Unified Parkinson’s Disease Rating Scale; H & Y, Hoehn and Yahr Stage; R, rigidity; B, bradykinesia; T, tremor; PI, postural instability; DBS STN, deep brain stimulation of the subthalamic nucleus; unk, unknown; ^1^ NM_032409.3, ^2^ NM_004562.3, ^3^ for both MDS-UPDRS and H & Y.

## Data Availability

The data presented in this study are available on request from the corresponding author. The data are not publicly available due to patient confidentiality.
